# Tocilizumab, a Proposed Therapy for the Cachexia of Interleukin6-Expressing Lung Cancer

**DOI:** 10.1371/journal.pone.0102436

**Published:** 2014-07-10

**Authors:** Katsutoshi Ando, Fumiyuki Takahashi, Motoyasu Kato, Norihiro Kaneko, Tokuhide Doi, Yuichiro Ohe, Fumiaki Koizumi, Kazuto Nishio, Kazuhisa Takahashi

**Affiliations:** 1 Division of Respiratory Medicine, Juntendo University Faculty of Medicine and Graduate School of Medicine, Bunkyo-Ku, Tokyo, Japan; 2 Department of Respiratory Internal Medicine, Kameda Medical Center, Kamogawa-City, Chiba, Japan; 3 Fukuoka Clinic, Adachi-Ku, Tokyo, Japan; 4 Department of Thoracic Oncology, National Cancer Center Hospital East, Kashiwa, Chiba, Japan; 5 Shien-lab, National Cancer Center Hospital, Chuo-ku, Tokyo, Japan; 6 Department of Genome Biology, Kinki University Faculty of Medicine, Higashiosaka City, Osaka, Japan; Ospedale Pediatrico Bambino Gesu’, Italy

## Abstract

**Background:**

We previously reported the role of IL-6 in a murine model of cancer cachexia and currently documented a patient in whom tocilizumab, anti-IL-6 receptor antibody, dramatically improved cachexia induced by IL-6 over-expressing lung cancer. Despite this potential to alleviate cancer cachexia, tocilizumab has not been approved for this clinical use. Therefore, preceding our planned clinical trial of tocilizumab, we designed the two studies described here to evaluate the levels of IL-6 in patients with lung cancer and the effect of tocilizumab in a murine model of human cancer cachexia.

**Methods:**

First, we measured serum IL-6 levels in patients with lung cancer and analyzed its association with cachexia and survival. Next, we examined the effect of a rodent analog of tocilizumab (MR16-1) in the experimental cachexia model.

**Results:**

Serum IL-6 levels were higher in patients with cachexia than those without cachexia. In patients with chemotherapy-resistant lung cancer, a high IL-6 serum level correlated strongly with survival, and the cut-off level for affecting their prognosis was 21 pg/mL. Meanwhile, transplantation of IL-6-expressing Lewis Lung Carcinoma cells caused cachexia in mice, which then received either MR16-1 or 0.9% saline. Tumor growth was similar in both groups; however, the MR16-1 group lost less weight, maintained better food and water intake and had milder cachectic features in blood. MR16-1 also prolonged the survival of LLC-IL6 transplanted mice (36.6 vs. 28.5 days, p = 0.016).

**Conclusion:**

Our clinical and experimental studies revealed that serum IL-6 is a surrogate marker for evaluating cachexia and the prognosis of patients with chemotherapy resistant metastatic lung cancer and that tocilizumab has the potential of improving prognosis and ameliorating the cachexia that so devastates their quality of life. This outcome greatly encourages our clinical trials to evaluate the safety and efficacy of tocilizumab treatment for patients with increased serum IL-6.

## Introduction

Cancer cachexia is a hypercatabolic state characterized by patients’ reduction in muscle and fatty tissue as well as anorexia, asthenia and anemia. This condition is frequent in patients with advanced cancer and accompanied by a deteriorating quality of life and shortened survival time [1]–[5]. These changes appear to be part of an inflammatory response that includes the elaboration of proinflammatory cytokines such as interlukin-6 (IL-6) and tumor necrosis factor-alpha (TNF-α) [6]–[8]. Also in a study of lung cancer, patients with cachexia had heightened inflammatory responses manifested as increases of serum IL-6, soluble TNF-α receptor and C-reactive protein (CRP) [9]. Accordingly, the management of these cytokines is vital as is the palliation of symptoms and reduction in distress when treating patients with cancer cachexia.

IL-6, which is a multifunctional cytokine involved in the inflammatory and immunologic responses that are characteristic of many autoimmune diseases, is considered to be a key mediator of cachexia [10], [11]. To elucidate the role of IL-6 in cachexia induced by lung cancer, we previously established a lung cancer cell line transfected with IL-6 cDNA and transplanted these cells into C57BL/6 mice [12]. As a result, IL-6 induced marked cachectic changes in these mice including shortened survival times. Comparatively, results in mice treated with a tocilizumab analog that diminished the symptoms and lengthened lifespans suggested that this treatment specifically targets cancer cachexia [12]. Furthermore, we currently experienced and described a patient in whom tocilizumab, anti-IL-6 receptor antibody, dramatically alleviated cachexia induced by IL-6 over-expressing lung cancer [13]. This patient, whose diagnosis was large cell carcinoma with cancer cachexia, suffered a performance status (PS) that deteriorated to an ECOG score of 4 with an increase of serum IL-6 to 173 pg/ml. However, once IL-6 targeting treatment with tocilizumab began, his cachectic symptoms, PS and albumin levels rapidly improved, and a nine-month survival period followed without cachexia-related symptoms [13]. That outcome and our previous reports indicate that tocilizumab, which has been used mainly for the treatment of rheumatoid arthritis, systemic juvenile idiopathic arthritis and Castleman’s disease, has good potential as therapy for cancer cachexia, but it has not yet been approved. Accordingly, to evaluate the efficacy and safety of tocilizumab, we are currently planning the clinical trial.

MR16-1, a rat anti-murine IL-6 receptor antibody, has been characterized as a suitable rodent analog of tocilizumab [14], [15]. Previously, Mori et al. reported that MR16-1 prevented the development of cancer-related anemia in an experimental model, LC-06 JCK mice [16]. However, whether MR16-1 suppresses such cachectic changes as weight and appetite loss remains unclear and should be assessed before the clinical trial. Therefore, as a preliminary step, we have determined here whether serum IL-6 is a reliable surrogate marker for use in patients with lung cancer and cachexia, and verified the efficacy of MR16-1 in our previously established experimental model of that condition.

## Materials and Methods

### Data collection for the evaluation of serum IL-6 levels in patients with lung cancer

During the period April 2010 to March 2011, peripheral blood was drawn from 33 patients with lung cancer who were diagnosed by pathological testing at Kameda Medical Center, a 1000-bed tertiary care center. The serum was separated and stored at −70°C until analysis. We excluded patients who did not consent or whose condition would interfere with the study results, e.g., infectious disease or coexisting illness. As data measurement from blood samples, we obtained their verbal informed consent and documented in their clinical records. Our retrospective review, verbal consent procedure and analysis of their data in his study were approved by the Ethical Committee of the Kameda Medical Center.

### Evaluation of cachectic parameters in patients with lung cancer

First, to assess the patients’ clinical characteristics, we designated two groups representing the presence or absence of cancer cachexia at the time of serum sampling. The cachexia group and non-cachexia group were then compared in terms of baseline characteristics and factors in their blood samples. The diagnosis of cancer cachexia was established based on the published international consensus, which was weight loss greater than 5%, or weight loss greater than 2% in individuals already showing depletion according to current bodyweight and height [body-mass index (BMI) less than 20 kg/m^2^] or degenerated loss of skeletal muscle (sarcopenia) [1]. Next, to evaluate the relationship between these parameters and survival in cachectic patients with metastatic lung cancer, we selected 19 patients with stage IIIb or IV cancer whose supportive care had been received only after IL-6 evaluation. We then analyzed the correlation between patients’ characteristics and survival time. Finally, we calculated the most appropriate cut-off level of serum IL-6 for their survival.

### Cell lines and cultures with animal models of cancer cachexia

Lewis lung cancer (LLC) cells originated as a spontaneous carcinoma of the lung in a C57BL/6 mouse [17], [18]. We established LLC cells transfected with the human IL-6 gene (LLC-IL6), which has a potent ability to induce cancer cachexia in mice, as previously described [12]. Briefly, human IL-6 cDNA was introduced into Sal I site of the eukaryotic cDNA expression vector BMGNeo, and was transfected into LLC cells using the Lipofectin reagent (Gibco BRL, Gaithersburg, MD) according to the manufacturer’s instructions [12], [19]. Cells were maintained in Iscove’s Modified Dulbecco’s Medium (IMDM, Wako, Osaka, Japan) with 20% fetal bovine serum, penicillin and streptomycin (100 U/mL and 100 µg/mL, respectively).

### Murine models of cachexia

Virus-free 5-week-old male C57BL/6J mice were obtained from Japan SLC (Hamamatsu, Japan). The animals were group-housed (5 per cage) at a temperature of 24±2°C, relative humidity of 55%±10%, and a 12 h light/12 h dark cycle. After habituation for 1 week, mice were housed two per cage throughout the experiment. Chlorinated water and irradiated food were provided *ad libitum*. All procedures performed on the animals were approved by the institutional animal care and use committee (IACUC) of Juntendo University.

We previously established the experimental cachexia model by LLC-IL6 transplantation into 6-week-old mice [12]. Furthermore, in many other studies that determined the pathogenesis of cancer cachexia and pharmacological efficacy, mice were used at five to six weeks of age for the transplantation of cancer cell lines, as reported [16], [20], [21]. Accordingly, also in this study, LLC-IL6 (1×10^6^) was inoculated subcutaneously into the right flank of each mouse at 6-week-old after a foregoing period of housing, and we designated the day of LLC-IL6 inoculation as day 0.

### MR16-1 treatment protocol

MR16-1 (Low. No. MC45038) is a rat anti-murine IL-6 receptor antibody, and was kindly supplied from the laboratory of Chugai Pharmaceutical Co. (Kamakura, Japan). As presented in the Introduction, Mori et al. previously determined the efficacy of MR16-1 for cancer-related anemia when injected intraperitoneally in a once-weekly dose of 20 mg/kg [16]. On the other hand, we previously evaluated in detail cachectic parameters at twenty-one days after tumor inoculation and also confirmed that our experimental cachexia model with LLC-IL6 produced survival of approximately three weeks without any treatment [12]. Furthermore, in the clinical situation, tocilizumab usually is administered every two or four weeks with the same interval for the treatment of rheumatoid arthritis (RA) and Castleman disease [22], [23]. Similarly, we started MR16-1 treatment at a once-weekly dose of 20 mg/kg beginning at day 3 so as to administer it with the same interval and evaluated the cachectic parameters at twenty-one days after tumor inoculation.

### Evaluation of MR16-1 effect on cachecia and survival

First, to evaluate the efficacy of MR16-1 on cachexia, twenty mice were assigned to four groups. Mice in group 3 and 4 represented the cancer cachexia model in which LLC-IL6 was inoculated subcutaneously (cancer cachexia group), while groups 1 and 2 received only the same amount of saline solution, 0.9% sodium chloride (healthy control groups). Additionally, groups 2 and 4 received MR16-1 treatment as in the above protocol (labeled “with MR16-1”), and groups 1 and 3 received the same amount of saline solution (labeled “without MR16-1”).

We assessed body weight, food and water intake and tumor volume twice a week as previously described [20]. Tumor volume in groups 3 and 4 was calculated by (length)×(width)^2^/2 (cm^3^) [24]. Twenty-one days after tumor inoculation, the mice were anesthetized with somnopentyl and euthanized. At the time of sacrifice, we collected blood samples, then dissected and weighed muscles from the extremities and fat tissue around the testes [25], [26]. To determine the nutritional status of these animals, we measured levels of glucose and triglycerides in sera and assessed white blood cell (WBC) count, hematocrit (Ht), and platelet (Plt) counts in blood. Each group consisted of five mice, and all experiments were performed twice.

Next, to determine the effect of MR16-1 on survival, twenty mice were also transplanted with LLC-IL6 and randomly divided into two groups: one group received MR16-1 with the same protocol as above, and the second group received only the same amount of saline solution. Each group consisted of ten mice, and their survival time was analyzed after tumor inoculation. Weekly treatment had been continued with the same interval until death was confirmed.

### Measurement of cachectic parameters in blood

For the evaluation of patients’ data in our clinical study, serum IL-6 levels were determined using commercially available enzyme-linked radioimmunoassay and enzyme-linked immunosorbent assay (ELISA) kits according to the manufacturers’ instructions (SRL Inc., Tokyo, Japan). Other parameters were also measured by routine examination at Kameda Medical Center. Additionally, to evaluate the efficacy of MR16-1 in the experimental animals, serum IL-6 was measured by using a murine IL-6 ELISA Kit, and all other parameters were measured using routine laboratory tests (KAC Inc., Kyoto, Japan).

### Statistical analyses

We performed statistical analyses with SPSS version 20, and data are expressed as means ± standard deviation. The Chi-squared test or Mann-Whitney test was used as appropriate to compare patients’ characteristics in our clinical study. Correlations of patients’ characteristics with survival were evaluated by Spearman’s rank correlation analysis. To determine the most appropriate cut-off level of serum IL-6 for one month survival, we used Akaike’s information criterion (AIC), which is an indicator for evaluating the goodness of fit for a statistical model [27], [28]. A smaller AIC value indicated a more reliable model for predicting outcomes. Survival rates were estimated with the Kaplan-Meier method using the cut-off value calculated by AIC analysis.

To evaluate the efficacy of MR16-1, we compared variables for the two groups using an unpaired *t*-test or Tukey’s Honest Significant Differences after analysis of variance (ANOVA). The survival curve of each group was analyzed by log rank test. For all statistical analyses, a p value less than 0.05 was considered significant.

## Results

### Clinical profile of 33 patients with lung cancer

As Table 1 shows, among the 33 patients profiled in this clinical study, the mean age was 71.3±10.0 years, and 76% were males. Seventeen of the patients had been diagnosed with adenocarcinoma (52%), thirteen with squamous cell carcinoma (39%), one with large cell carcinoma (3%) and two with small cell carcinoma (6%). A total of 26 patients had been treated with chemotherapy (n = 22), radiotherapy (n = 4) or surgery (n = 1) before or after evaluation of IL-6. Seven patients received supportive care only. After the evaluations for this study, 19 patients had supportive care only; 11 had a history of chemotherapy administration and one had radiotherapy. Eighteen patients were diagnosed with cancer cachexia, as established in the international consensus study [1].

**Table 1:** Patients’ characteristics and comparison between patients with or without cachexia.

–n (%)	All patients (n = 33)	Cachexia group (n = 18)	Non-cachexia group (n = 15)	p value
Age, y	71.3±10.0	74.2±10.4	67.9±8.5	0.067
Male/female sex, n	25/8	14/4	11/4	1.0
Weight, kg	53.7±8.8	50.3±7.5	57.1±9.0	0.050
Body mass index	20.7±3.1	19.7±2.0	21.7±3.8	0.227
Weight loss, %	5.2±6.7	10.3±6.0	0.5±2.8	<0.001
Histological status of tumor				
Adenocarcinoma	17 (52)	9 (50)	8 (53)	1.0
Squamous-cell	13 (39)	8 (44)	5 (33)	0.722
Large-cell	1 (3)	1 (6)	0	1.0
Small-cell	2 (6)	0	2 (13)	0.199
ECOG performance status				
0	9 (27)	0	9 (60)	<0.001
1	7 (21)	4 (22)	3 (20)	1.0
2	4 (12)	3 (17)	1 (7)	0.607
3	9 (27)	7 (39)	2 (13)	0.134
4	4 (12)	4 (22)	0	0.108
Disease stage				
IA/IB	0/1 (3)	0/0	0/1 (7)	0.455
IIA/IIB	0/4 (12)	0/2 (11)	0/2 (13)	0.199
IIIA/IIIB	3 (9)/3 (9)	2 (11)/2 (11)	1 (7)/1 (7)	1.0
IV	22 (67)	12 (67)	10 (67)	1.0
Blood tests				
White blood cell (/µL)	9,064±5,080	11,722±5,308	5,873±2,187	<0.001
Total protein (g/dL)	6.53±0.68	6.38±0.76	6.73±0.55	0.257
Albumin (g/dL)	3.05±0.64	2.70±0.53	3.47±0.50	<0.001
C reactive protein (mg/dL)	5.08±5.39	7.09±5.89	2.49±3.32	<0.01
IL-6 (pg/mL)	27.9±31.6	40.2±37.4	13.0±12.2	<0.001
Treatment –n (%)				
Chemotherapy	22 (67)	10 (56)	12 (80)	
Post evaluation of IL-6	11 (33)	2 (11)	9 (60)	<0.01
Operation	1 (3)	1 (6)	0	
Post evaluation of IL-6	1 (3)	1 (6)	0	1.0
Radiotherapy	4 (12)	3 (17)	1 (7)	
Post evaluation of IL-6	3 (9)	2 (11)	1 (7)	1.0
Supportive care only	7 (21)	5 (28)	2 (1)	
Supportive care after IL-6evaluation	19 (58)	14 (78)	5 (33)	<0.001

Plus-minus data are presented as means ± SD.

Patients’ characteristics in the cachexia group (n = 18) compared to the non-cachexia group (n = 15) are shown in Table 1. Mean age, gender, BMI, histological status and disease stage were not statistically different for the two groups. The cachexia group had a greater proportion of patients with a low performance status (ECOG score <2, 77.8% vs. 20.0%), and more of them were treated with supportive care after performance evaluation, (77.8% vs. 33.3%) than in the non-cachexia group. In the blood samples, WBC counts were higher in the cachexia group (11,722 vs. 5,873/µL), as were CRP content (7.1 vs. 2.5 mg/dL), and serum IL-6 levels (40.2 vs. 13.0 pg/mL); however, the cachexia group’s albumin levels were lower (2.7 vs. 3.5 g/dL) than in the non-cachexia group. Total protein was no different between the two groups (6.4 vs. 6.7 g/dL).

Our results resembled those in a previous large cohort of patients with pancreatic cancer studied by Bachmann et al [29]. Accordingly, although the number of patients included in our analysis was relatively small, the results assured that our data were valuable for more detailed analysis.

### Correlation of serum IL-6 levels with survival

We then correlated the cachectic parameters analyzed with survival in 19 patients with stage IIIB or IV cancer who received supportive care after IL-6 evaluation (Table 2). All those patients had been diagnosed with non-small cell lung cancer (NSCLC); ten with adenocarcinoma, eight with squamous cell carcinoma and one with large cell carcinoma. Survival times after that evaluation had a statistically significant negative correlation with ECOG performance status (r = −0.621, p = 0.005), CRP (r = −0.529, p = 0.020) and serum IL-6 levels (r = −0.639, p = 0.003), and a positive correlation with albumin levels (r = 0.588, p = 0.008). WBC counts (r = −0.425, p = 0.069) and total protein (r = 0.515, p = 0.060) also tended to correlate with prolonged survival, but not at the level of statistical significance. Meanwhile, serum IL-6 levels correlated well with albumin (r = −0.449, p = 0.054) and CRP (r = 0.543, p = 0.016), but not with total protein (r = −0.006, p = 0.981).

**Table 2:** Correlation between patients’ characteristics and survival or IL-6 levels.

	Patients (n = 19)	
	r	p value
*Survival time*		
Age	−0.227	0.351
ECOG performance status	−0.621	0.005
White blood cell (/µL)	−0.425	0.069
Total protein (g/dL)	0.515	0.060
Albumin (g/dL)	0.588	0.008
C reactive protein (mg/dL)	−0.529	0.020
IL-6 (pg/mL)	−0.639	0.003
*IL-6 levels*		
ECOG performance status	0.206	0.329
White blood cell (/µL)	0.314	0.190
Total protein (g/dL)	−0.006	0.981
Albumin (g/dL)	−0.449	0.054
C reactive protein (mg/dL)	0.543	0.016

We selected 19 patients with stage IIIB or IV and supportive care after evaluation, and analyzed the correlation between patients’ characteristics and survival time or IL-6 level. All those patients had been diagnosed with non-small cell lung cancer (NSCLC).

We also considered that it was important to include the analysis of patients who had been diagnosed with small cell lung cancer (SCLC) or who had been under treatment to determine the effect of IL-6 on chemotherapy. However, since the number of these patients was small, we could not verify its effect on chemotherapy or their prognosis in this study.

### Serum IL-6 levels and survival

**Figure 1:**
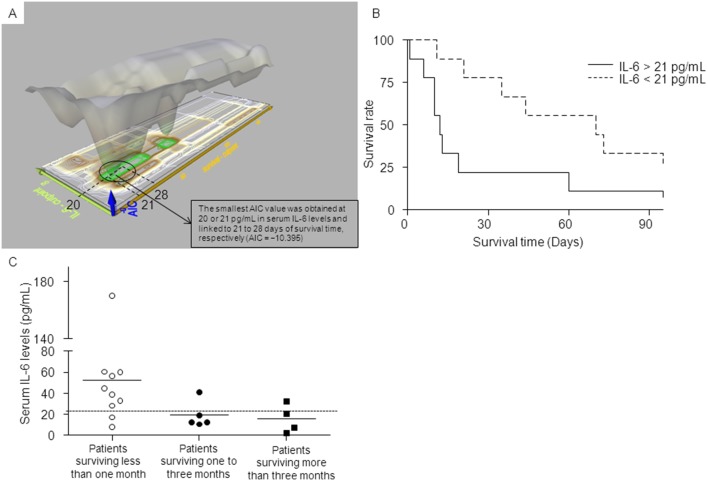
Relationship between serum IL-6 levels and survival. (**A**) A conservative threshold linked 20 or 21 pg/ml of IL-6 in serum (y-axis) with 21 to 28 days of survival time (x-axis), as determined by AIC analysis (see Methods), satisfactorily identifying the most appropriate cut-off level (z-axis, AIC = −10.395). Note that patients with serum IL-6 levels ≥21 pg/ml were predicted to have less survival time. (**B**) The probability of surviving one and three months after evaluation was lower in patients with IL-6 >21 pg/ml than those with <21 pg/ml (one month survival, 20.0% vs. 77.8%, p = 0.007; and three months survival 10.0% vs. 33.3%, p = 0.025). (**C**) The proportion of patients with IL-6 ≥21 pg/ml was 80.0% for patients surviving less than one month, 20.0% for patients surviving one to three months and 25.0% for patients surviving more than three months.

To determine the most appropriate cut-off level of patients’ serum IL-6 content and its relationship to survival, the AIC was calculated (Figure 1A). As a result, the smallest AIC value was obtained at 20 or 21 pg/mL in serum IL-6 levels and linked to 21 to 28 days of survival time, respectively (AIC = −10.395, each combination was the same value). This revealed that patients with serum IL-6 levels ≥21 pg/ml could be predicted to have the lower survival time of one month or less.

Based on the AIC statistics, patients were then divided into those with IL-6 >21 pg/mL and <21 pg/mL and analyzed for the probability of survival using the Kaplan–Meier method. Figure 1B reveals the survival pattern after IL-6 evaluation. There the probability of survival for one and three months after evaluation is clearly lower in patients with IL-6 >21 pg/mL than with <21 pg/mL (one month survival, 20.0% vs. 77.8%, p = 0.007; and three months survival 10.0% vs. 33.3%, p = 0.025). We further classified patients into three groups according to survival time: within one month, one to three months and over three months. Analysis of serum IL-6 levels in each group, as Figure 1C depicts, indicated that the proportion of patients with IL-6 ≥21 pg/mL was 80.0% who survived within the one-month time frame, whereas 20.0% survived for one to three months, and 25.0% survived over three months.

### The effect of MR16-1 on cancer cachexia in LLC-IL6-bearing mice

At day 21 of our duplicate experiments, a total of eight in ten mice survived in each cancer cachexia group (groups 3 and 4), while all ten mice survived in healthy control groups (groups 1 and 2). We sacrificed a total of 36 mice, and their cachectic parameters are shown in Table 3.

**Table 3:** Cachectic parameters in control and treated mice at day 21.

Parameters	Healthy control groups		Cancer cachexia group	
	Group 1 (n = 10)without MR16-1	Group 2 (n = 10)with MR16-1	Group 3 (n = 8)without MR16-1	Group 4 (n = 8)with MR16-1
Carcass weight (g)	25.9±1.3	26.0±1.5	22.1±0.9*	24.1±2.3****
Gastrocnemius muscle (mg)	128.1±49.2	115.4±32.2	60.4±29.3*	106.6±22.8***
Quadriceps muscle (mg)	102.7±46.5	114.2±33.6	14.7±8.6*	48.5±21.2***
Biceps femoris muscle (mg)	145.4±27.9	174.6±85.3	27.1±12.3*	59.4±28.9***
Fat tissue around testis (mg)	490.5±80.8	468.4±70.7	169.4±48.1*	312.4±90.3****
White blood cell (/µL)	4,667±2,317	3,867±1,892	48,350±18,288*	4,100±880***
Hematocrit (%)	32.8±2.5	35.4±1.2**	9.4±4.4*	21.8±2.1***
Platelet (×10^4^/µL)	54.8±25.7	69.3±20.9	102.2±28.1**	68.5±28.5
Triglyceride (mg/dL)	87.0±18.2	81.2±28.1	23.0±9.1*	48.0±14.4***
Glucose (mg/dL)	311.6±174.9	260.0±40.6	29.6±9.5*	101.0±36.0***

In cancer cachectic groups (groups 3 and 4), Lewis Lung Carcinoma (LLC) cells transfected with IL-6 cDNR (LLC-IL6) were implanted subcutaneously into a total of 20 C57BL/6J mice. We administered MR16-1, anti-murine IL-6 receptor monoclonal antibody, intraperitoneally at a dose of 20 mg/kg once a week into 10 mice (Group 4), while 0.9% saline into 10 mice (Group 3), respectively.

Eight mice in both the MR16-1 and control groups survived for 21 days after the tumor inoculation. After sacrifice, these mice were evaluated for cachectic parameters. Carcass weight was weight without tumor.

Data represent mean ± S.D. Statistical significance was evaluated with Turkey’s test after a one-way ANOVA.

* Significantly different from group 1 (tumor (−), MR16-1 (−)); p<0.01.

** Significantly different from group 1 (tumor (−), MR16-1 (−); p<0.05.

*** Significantly different from group 3 (tumor (+), MR16-1 (−)); p<0.01.

**** Significantly different from group3 (tumor (+), MR16-1 (−)); p<0.05.

As for comparison between untreated mice of the healthy control (group 1) and cancer cachexia group (group 3), carcass weights (group 1, 25.9 g vs. group 3, 22.1 g, p<0.001), weights of gastrocnemius muscle (128.1 mg vs. 60.4 mg, p = 0.006), quadriceps muscle (102.7 mg vs. 14.7 mg, p<0.001), biceps femoris muscle (145.4 mg vs. 27.1 mg, p<0.001), and fat tissue around the testes (490.5 mg vs. 169.4 mg, p<0.001) were significantly higher in group 1 than group 3. The values of Ht (32.8 vs. 9.4%, p = 0.001), triglyceride (87.0 vs. 23.0 mg/dL, p<0.001) and glucose (311.6 vs. 29.6 mg/dL, p<0.001) were also higher in group 1 than those in group 3, respectively. Since these results were similar to those in our previous report [12], we confirmed the reproducibility of our established experimental model.

As for the comparison between treated and untreated cancer cachexia mice (group 3 [without MR16-1] and 4 [with MR16-1]), on the other hand, carcass weights (group 3, 22.1 g vs. group 4, 24.1 g, p = 0.032), weights of gastrocnemius muscle (60.4 mg vs. 106.6 mg, p<0.001), quadriceps muscle (14.7 mg vs. 48.5 mg, p = 0.007), biceps femoris muscle (27.1 mg vs. 59.4 mg, p = 0.001), and fat tissue around the testes (169.4 mg vs. 312.4 mg, p = 0.015) were significantly higher in group 4 than group 3. In addition, the WBC (48,350 vs. 4,100/µL, p = 0.004) count was significantly lower, and the values of Ht (9.4 vs. 21.8%, p = 0.004), triglyceride (23.0 vs. 48.0 mg/dL, p = 0.005), and glucose (29.6 vs. 101.0 mg/dL, p = 0.003) were higher in group 4 than those in group 3, respectively (Table 3). Meanwhile, serum IL-6 levels were higher in group 4 than group 3 (41.9±17.9 vs. 296.1±283.2 pg/mL, p<0.001).

**Figure 2:**
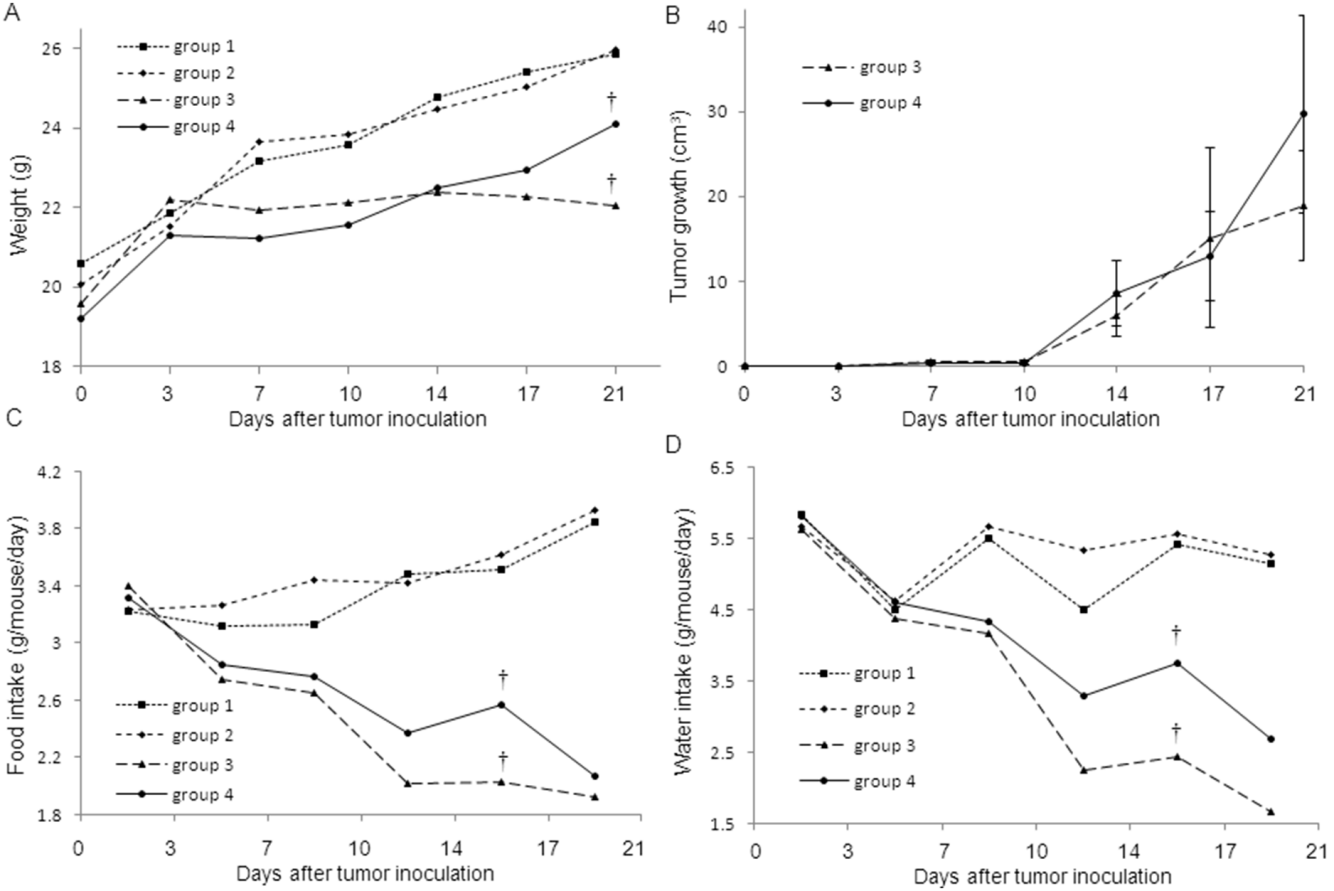
Effects of MR16-1 on carcass weight (a), tumor growth (b), food intake (c), and water intake (d). Transplantation of IL-6-expressing LLC cells caused cachexia in mice (groups 3 and 4), while groups 1 and 2 received only the same amount of saline solution, 0.9% sodium chloride (healthy control groups). We administered MR16-1 (groups 2 and 4) or 0.9% saline (groups 1 and 3), respectively. Body weight, tumor size and food and water intake were measured on days 0, 3, 7, 10, 14, 17 and 21 after transplantation. The measured quantity of food or water was divided by the number of mice and days to determine each intake per animal per day. Statistical significance was evaluated with Tukey’s Honest Significant Differences (HSD) after the one-way ANOVA. Data represent mean and S.D. (S.D. data of weight are shown in Table 4). Tumor growth was not significantly different between groups 3 and 4 (B), but body weight (A) and food (C) and water intake (D) were significantly improved in group 4 (†, p<0.01). No significant differences were observed in the carcass weight and food and water intakes between the untreated (group 1) and treated healthy mice (group 2).

Figure 2 shows serial changes of body weight (A), tumor growth (B), and food (C) and water (D) intake. As for the comparison between cancer cachexia groups, tumor growth was not statistically significantly different (Figure 2B, p = 0.061), but body weights remained higher in group 4 (Figure 2A, p = 0.032). Progressive reductions in food and water intake were observed in both groups after inoculation of LLC-IL-6, but MR16-1 tended to improve these values to the point that significant differences were prominent at day 14 (Figures 2C, p<0.001, and Figure 2D, p<0.001). These results indicate that administration of MR16-1 significantly decreased the reduction of carcass weight, food and water intakes without affecting tumor growth in a murine model of cachexia.

### Safety of MR16-1 in healthy control mice

To determine the safety of MR16-1, we also evaluated other biochemical parameters in healthy control mice and compared them between groups 1 and 2. As shown in Tables 3 and 4, biochemical parameters were not statistically significantly different between these two groups. The counts of WBC (4,667 vs. 3,867/µL, p = 0.8644) and platelets (548,000 vs. 693,000/µL, p = 0.249) were also not significantly different, but the values of Ht (32.8 vs. 35.4%, p = 0.049) was higher in group 2 than in group 1. Serial changes of body weight and intakes of food and water were not different between the two groups (Figure 2).

**Table 4:** Safety of MR16-1 in healthy control mice.

Parameters	Group 1 (n = 10) without MR16-1	Group 2 (n = 10) with MR16-1	p value
AST (IU/L)	54.6±27.3	42.3±6.3	0.905
ALT (IU/L)	40.0±27.4	34.3±4.3	0.730
LDH (IU/L)	304.0±124.6	221.3±45.8	0.190
Total bilirubin (mg/dL)	0.04±0.01	0.03±0.02	0.548
Alkaline phosphatase (IU/L)	379.4±50.1	385.6±51.8	1.0
Blood urea nitrogen (mg/dL)	28.8±3.6	30.7±2.8	0.421
Creatinine (mg/dL)	0.12±0.04	0.12±0.05	1.0
Uric acid (mg/dL)	1.16±0.45	1.00±0.52	0.690

To evaluate the safety of MR16-1, we appraised other biochemical parameters in the blood of healthy control mice. Group 2 received MR16-1 treatment, whereas group 1 received the same amount of saline solution.

Abbreviations used are: AST, aspartic acid aminotransferase; ALT, alanine aminotransferase; LDH, lactate dehydrogenase.

### The effect of MR16-1 on survival in LLC-IL6-bearing mice

**Figure 3:**
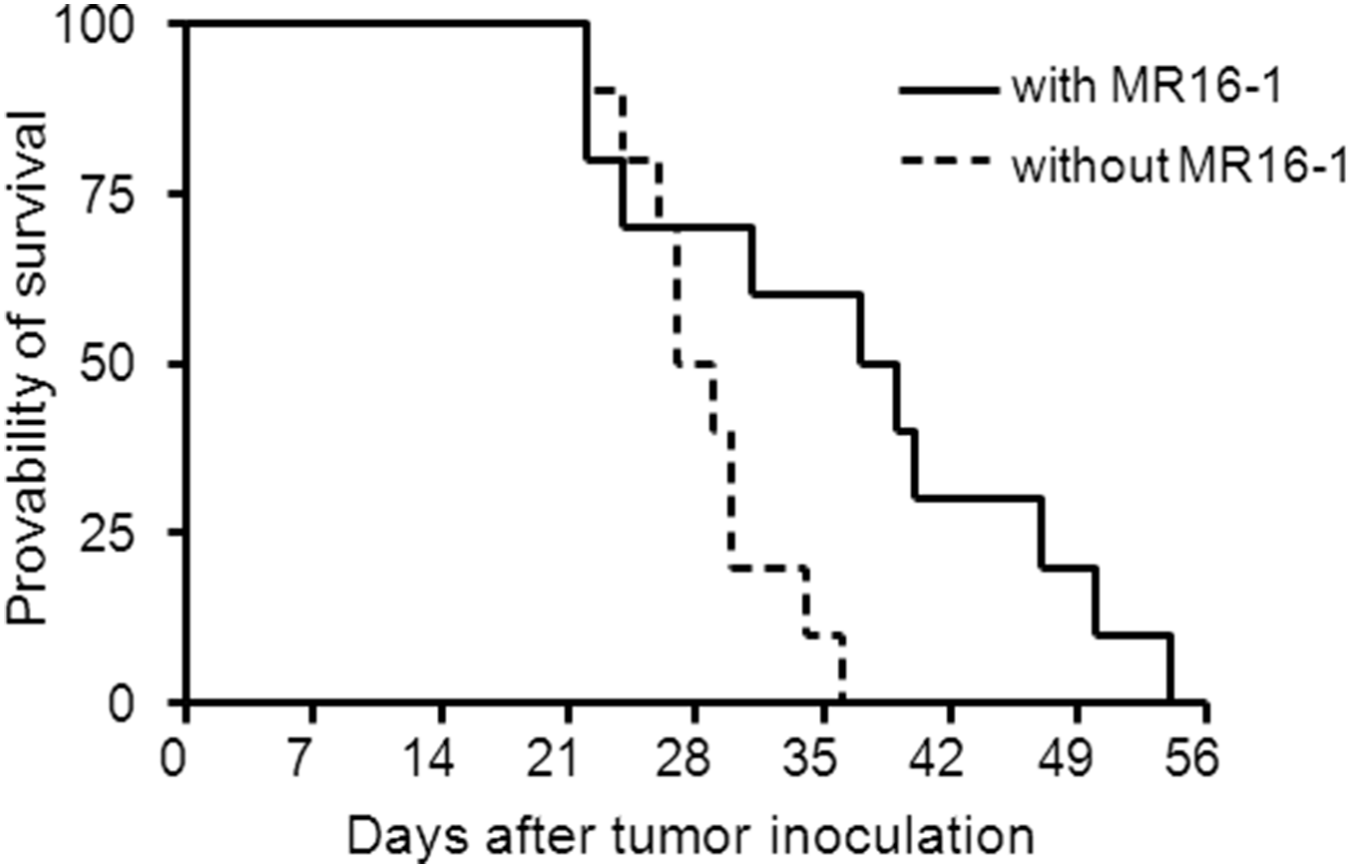
Effect of MR16-1 on survival of LLC-IL6 transplanted mice. Each group consisted of ten LLC-IL6 transplanted mice. One group received MR16-1 (solid line), and the other group received only the same amount of saline solution (dotted line). Weekly treatment had been continued with the same interval until death was confirmed. Mean ± S.D. of the survival of LLC-IL6 transplanted mice without MR16-1 was 28.5±4.1 days. The injection of MR16-1 prolonged the survival of LLC-IL6 transplanted mice to 36.6±11.1 days (p = 0.016).

Twenty mice were transplanted with LLC-IL6 cells and divided into two groups; with (treated) or without MR16-1 (untreated). Survival time was determined as shown in Figure 3. Survival times of treated and untreated mice were 36.6±11.1 and 28.5±4.1 days, respectively, and MR16-1 significantly improved the overall survival time after tumor inoculation (p = 0.016). These results indicate that MR16-1 has the potential to improve survival as well as suppress the progression of cancer cachexia.

## Discussion

In our clinical and *in vivo* studies, we showed the usefulness of serum IL-6 as a surrogate marker for evaluating the prognosis and efficacy of anti-IL-6 receptor antibody as a mediator of cancer cachexia. We found that (1) the presence of serum IL-6 correlated strongly with survival in patients bearing chemotherapy-resistant metastatic lung cancer; (2) patients with serum IL-6 levels ≥21 pg/mL were predicted to have shorter survival times; (3) MR16-1 had the beneficial effect of ameliorating cancer cachexia and survival. Accordingly, we showed for the first time that IL-6 was a useful, well-targeted treatment for chemotherapy-resistant metastatic lung cancer, and tocilizumab seemed to be a promising and novel anti-cachectic agent for the treatment of patients with IL-6 over-expressing tumors.

At present, the association of biochemical markers with prognosis has been widely reported in patients with metastatic cancer. That is, high CRP levels indicate these patients’ systemic inflammatory reaction and relate to their typically low skeletal muscle index and serum albumin concentration as well as poor prognosis [30]–[32]. Some studies reported that cut-off levels of CRP and albumin for affecting prognosis were 10 mg/L and 3.5 g/dL, respectively [33]–[35]. Since these reactions result from the effect of cytokines, our results also indicate that serum IL-6 levels logically bear a strong correlation with survival times in patients with chemotherapy-resistant metastatic lung cancer. Furthermore, we found that a serum IL-6 level of 21 pg/mL is a conservative threshold for affecting their prognosis. This cut-off point was based on the data in this study, but we consider that it could be a more accurate indicator than CRP and albumin.

As for the treatment of cancer cachexia, of the several agents evaluated so far, corticosteroids and progestational agents have been the best shown to alleviate cancer-related anorexia in prospective trials and a meta-analysis [36]–[38]. However, no agent that improves life span or quality of life has ever been documented. Therefore, establishing a new therapeutic modality to alleviate cancer cachexia is urgently needed. In that context, we previously reported that tocilizumab had remarkably ameliorated a patient’s cancer cachexia induced by an IL-6 over-expressing lung cancer [13]. Although this patient’s tumor had gradually progressed, his overall condition had stabilized, and he survived nine months after starting tocilizumab treatment [13]. Similarly, in experimental animal models, MR16-1 decreased the progression of cachexia. Moreover, serum IL-6 levels increased in a MR16-1-treated group compared to an untreated control group. In regard to these mechanisms, Nishimoto et al. analyzed the kinetics of serum IL-6 and soluble IL-6 receptor (sIL-6R) as well as the proportion of sIL-6R saturated with tocilizumab after its administration to patients with RA and Castleman disease. The authors reported that sIL-6R increased markedly as IL-6 did. That is, the administration of tocilizumab led to the formation of tocilizumab/sIL-6R immune complexes thereby prolonging the elimination half-life of sIL-6R. The subsequent unavailability of tocilizumab-free IL-6R then inhibited the IL-6R-mediated consumption of IL-6 [39]. Our results agree with those responses in a previous study and in our reported case [13], [39]. Accordingly, we believe that tocilizumab is useful for the treatment of cachexia induced by IL-6 over-expressing cancer.

Meanwhile, we have also considered some concerns regarding an inhibitory role of IL-6. First, some cytokines are known to participate in anti-tumor immunity by activating or regulating the function of antitumor effector T cells [40]–[42]. Therefore, blocking IL-6 signaling might promote the growth and differentiation of tumor cells by inhibiting anti-tumor immunity. To the contrary, our results revealed that tumor growth was not statistically different, despite a modest increase in MR16-1-treated mice than in the untreated control group. Furthermore, in our previous case report, although the tumor was progressive, the cachectic condition decreased substantially [15]. However, Narita et al. demonstrated that a blockade of IL-6 signaling by anti-IL6 receptor antibody exerted an antitumor effect through the down-modulation of arginase-1 activity and up-regulation of MHC class II expression on tumor-associated CD11c+ dendritic cells [43]. That is, its effect on a cachectic condition was promising, but the potential effect on tumor growth remained controversial. Second, a variety of side effects have been reported with respect to tocilizumab treatment [44]. In the clinical trials, more than 10% of patients reportedly had upper respiratory tract infections, and many had decreased neutrophil counts [44]. Our study of mice *in vivo* revealed that the MR16-1-treated group had WBC counts within normal, but extremely lower than in the control group. Yet the neutrophil count of our previous patient did not decline below 7,000 K/mm^3^, and no evidence of infection requiring treatment emerged [45]. However, we will carefully evaluate the factors encountered in these experiments as we proceed with related clinical trials. Finally, in our experimental study, we used 6-week-old young mice that were sexually immature and in a phase of rapid body growth. Although many other previous reports used mice at five to six weeks [16], [20], [21], we did not confirm if experiments are performed in more mature mice. This point was our limitation of this study and we should evaluate it in further study.

In conclusion, our clinical and experimental studies revealed that serum IL-6 is a surrogate marker for evaluating the prognosis of patients with chemotherapy resistant metastatic lung cancer and that tocilizumab has the potential of ameliorating the cachexia as well as preventing its progression that so devastates their quality of life. This outcome greatly encourages our clinical trials to evaluate the safety and efficacy of tocilizumab treatment for patients with increased serum IL-6.
